# Solving the inverse problem of time independent Fokker–Planck equation with a self supervised neural network method

**DOI:** 10.1038/s41598-021-94712-5

**Published:** 2021-07-30

**Authors:** Wei Liu, Connie Khor Li Kou, Kun Hee Park, Hwee Kuan Lee

**Affiliations:** 1 Bioinformatics Institute, Agency for Science, Technology and Research (A*STAR), 30 Biopolis Street, #07-01 Matrix, Singapore, 138671 Singapore; 2 Centre for Quantum Technologies, National University of Singapore, 3 Science Drive 2, Singapore, 117543 Singapore; 3 School of Computing, National University of Singapore, 13 Computing Drive, Singapore, 117417 Singapore; 4 Singapore Eye Research Institute (SERI), 11 Third Hospital Ave, Singapore, 168751 Singapore; 5 Image and Pervasive Access Laboratory (IPAL), 1 Fusionopolis Way, #21-01 Connexis (South Tower), Singapore, 138632 Singapore; 6 Rehabilitation Research Institute of Singapore, 11 Mandalay Road #14-03, Clinical Sciences Building, Singapore, 308232 Singapore; 7 Singapore Institute for Clinical Sciences, A*STAR, 30 Medical Drive, Singapore, 117609 Singapore

**Keywords:** Mathematics and computing, Physics

## Abstract

The Fokker–Planck equation (FPE) has been used in many important applications to study stochastic processes with the evolution of the probability density function (pdf). Previous studies on FPE mainly focus on solving the forward problem which is to predict the time-evolution of the pdf from the underlying FPE terms. However, in many applications the FPE terms are usually unknown and roughly estimated, and solving the forward problem becomes more challenging. In this work, we take a different approach of starting with the observed pdfs to recover the FPE terms using a self-supervised machine learning method. This approach, known as the inverse problem, has the advantage of requiring minimal assumptions on the FPE terms and allows data-driven scientific discovery of unknown FPE mechanisms. Specifically, we propose an FPE-based neural network (FPE-NN) which directly incorporates the FPE terms as neural network weights. By training the network on observed pdfs, we recover the FPE terms. Additionally, to account for noise in real-world observations, FPE-NN is able to denoise the observed pdfs by training the pdfs alongside the network weights. Our experimental results on various forms of FPE show that FPE-NN can accurately recover FPE terms and denoising the pdf plays an essential role.

## Introduction

The Fokker–Planck equation (FPE) is an important tool to study stochastic processes commonly used to model complex systems. The time evolution of variables in a stochastic process is affected by random fluctuations which makes it impossible to derive a deterministic trajectory. However, the randomness can be accounted for by characterizing the process with the probability density function (pdf) of the random variable. As a class of partial differential equations (PDEs), FPE governs how the pdf evolves under drift and diffusion forces. FPE has a wide range of applications in many disciplines. For example, the master equation can be approximated by FPE which is easier to be solved numerically^1^. FPE has been used to link the Langevin equation to Monte Carlo (MC) methods in stochastic micromagnetic modeling, where the drift and diffusion terms of FPE derived from the MC method have been proven to be analytically equivalent to the stochastic Landau–Lifshitz–Gilbert (LLG) equation of Langevin-based micromagnetics^2^. The eigenfunctions of FPE have been used to approximate the eigenvectors of the graph Laplacian, providing a diffusion-based probabilistic interpretation of spectral clustering^3^. Beyond theory, FPE is also a common tool to model real-world phenomena. For example, it has been used to determine the first passage time from one protein conformation to another^4^, to describe the weights of deep networks when updating them using stochastic gradient descent^5^, to characterize the behavior of driver-assist vehicles in freeway traffic^6^ and to model the distribution of the personal wealth in socio-economics^7^. In all these applications on real-world pdfs, the FPE terms are proposed first and then validated by comparing the calculated pdf with the experimental data, which can be seen as a forward problem. To date, many numerical approaches have been proposed to solve the forward problem of FPE which is to calculate the pdf with given drift and diffusion terms of FPE, including the finite element method^8–10^, the finite difference method^11,12^, the path integral method^13^, and the deep learning methods^14,15^.

Solving the forward problem in the case of FPE can often be challenging. For many complex systems, the form and parameters of FPE are unclear. For instance, in the field of socio-economics, the coefficient of money transaction is treated as a constant which may be an oversimplification^7^. In DNA bubble dynamics, the free energy terms have to be approximated^16^. As such, successful applications of forward modeling of FPE are limited to a few simple FPE forms. For example, the most commonly used FPE is derived from the Ornstein–Uhlenbeck process^17^ which has a linear drift term and a constant diffusion term. However, FPE can adopt more complicated forms. In such cases, forward modeling becomes a tedious trial-and-error process of proposing reasonable FPE terms and validating with experimental data. Alternatively, it will be more efficient to solve the inverse problem, which is to derive the terms of the FPE directly from the experimental data. Besides higher efficiency in many cases, the inverse problem can be seen as a data-driven approach for scientific discovery. It requires no prior knowledge except assuming the observed data satisfies FPE, enabling us to uncover the unknown mechanisms hidden behind the stochastic processes.

One of the conventional ways to solve the inverse problem of the FPE is linear least square (LLS). However, as shown in the Supplementary, the calculated FPE terms become error-prone when the observed pdf is noisy due to limited sample size. Machine learning techniques have been promising in solving various inverse problems. In particular, in recent years, neural network models have been used to learn to solve the inverse problem using observable data as the input and outputs. In the field of reinforcement learning and imitation learning, the value iteration network has been proposed^18^, where the network is trained on optimal policies to recover the approximate reward and value functions of a planning computation. To the best of our knowledge, there has been one prior work which uses neural networks to solve the inverse problem of FPE. Chen et al developed a general framework which takes the spatial and temporal variables as the input and outputs the corresponding probability density values^19^. FPE is incorporated into the network loss function based on the physics informed neural network (PINN) technique^20^, where the derivatives are calculated by automatic differentiation. In their approach, the random fluctuation causing the stochastic process is assumed to be the Brownian noise and/or the Lévy noise, and the coefficient of the fluctuations are constant. Chen’s work took the Lévy noise into consideration, which is a further generalization than most FPE studies which only consider the Brownian noise (usually called Gaussian white noise). However, the assumption that the noise coefficient is constant is much stricter than in a typical FPE. In actual applications, the random fluctuation may not be independent of the spatial variable and consequently the diffusion term becomes an unknown function.

More broadly, PDE-Nets have been proposed to uncover the underlying PDE models of complex systems in a supervised learning manner^21,22^. Unlike the PINN-based methods, PDE-Nets calculate the derivatives using a new and accurate finite difference method. However, the PDE-Nets do not specifically handle the noise within the experimental pdfs which is majorly due to limited sample size, and the noise is an inevitable source of error. Although the noise is partially removed in PDE-Nets by smoothing the pdfs with the Savitzky–Golay (SG) filter^23^, the remaining noise is still a major error source of the uncovered PDE.

In this paper, we propose a novel neural network model to solve the inverse problem of FPE. Specifically, we directly embed FPE into the neural network architecture, where the drift and diffusion terms are represented by the trainable network weights. Our network, named as the FPE-based neural network (FPE-NN), is trained in a self-supervised manner where the network is used to predict the distributions at neighboring time points based on the input distribution at a single time point. Additionally, to denoise the experimental pdfs, we train both the network weights (representing the FPE terms) and the input (representing the experimental pdfs) in an alternating way. Our FPE-NN has two key advantages over prior works. First, FPE-NN does not assume any form of the FPE terms, making it more flexible than the PINN-based method. Second, FPE-NN can denoise the pdfs in experimental data. In fact, we find that the denoising of experimental pdfs is essential to accurately recover the FPE terms. Our experiments on simulated data show that FPE-NN is able to recover unknown FPE terms, denoise the experimental pdfs, and predict future distributions accurately.

## Methods

In this work, we focus on the one-dimensional and time-independent FPE, whose form is shown below:

1 $$\begin{aligned} \frac{\partial}{\partial t}P(x,t)= \frac{\partial}{\partial x}(g(x)P(x,t)) + \frac{\partial^{2}}{\partial\,x^{2}}(h(x)P(x,t)) \end{aligned}$$

where $P: {\mathbb {R}} \times {\mathbb {R}} \rightarrow {\mathbb {R}}^+ \cup \{0\}$ is the pdf over the observable *x* and the time *t*, $g: {\mathbb {R}} \rightarrow {\mathbb {R}}$ is the drift term and $h: {\mathbb {R}} \rightarrow {\mathbb {R}}$ is the diffusion term. In actual implementation, *P*(*x*, *t*), $\partial P(x,t)/\partial t$, *g*(*x*), and *h*(*x*) are discretized over variable *x* and denoted as ${\varvec{P}}(t)$, ${\varvec{\partial }}_{\varvec{t}} {\varvec{P}}(t)$, ${\varvec{g}}$, and ${\varvec{h}}$, respectively. More specifically, we discretize variable *x* into a number of bins on the support, $(x_1, x_2, \ldots )$. Then P(x,t) becomes the vector ${\varvec{P}}(t) = (P(x_1,t), P(x_2,t), \ldots )$, and g(x) becomes the vector ${\mathbf {g}} = (g(x_1), g(x_2), \ldots )$. The same transform applies to all the other functions.

In order to solve the inverse problem, we focus on the scenario in which a pdf is assumed to satisfy FPE and the data within some time period is measured. We also assume *P*(*x*, *t*) is differentialble and has a finite support, which is satisfied by most real-world pdfs. However, the measured pdf, denoted as ${\varvec{P}}_{noisy}(t)$, is corrupted with multiplicative noise, and the exact forms of ${\varvec{g}}$ and ${\varvec{h}}$ are unknown. Hence, we propose to train a FPE-based neural network model FPE-NN in a self-supervised way, based on ${\varvec{P}}_{noisy}(t)$ only, to find ${\varvec{g}}$, ${\varvec{h}}$ and the true pdf which is denoted as ${\varvec{P}}_{clean}(t)$. We currently focus on the one-dimensional FPE and will expand to multi-dimensional FPE in future work.

In the following sections, we first give an overview of how to find ${\varvec{g}}$ and ${\varvec{h}}$ and to recover ${\varvec{P}}_{clean}(t)$ in an alternating training process. Next, we introduce the architecture of the network FPE-NN. This is followed by training details such as initialization, alternating training steps, stopping criterion, and an application of FPE-NN for predicting future distributions.

### Overview of the FPE-NN training process

**Figure 1:**
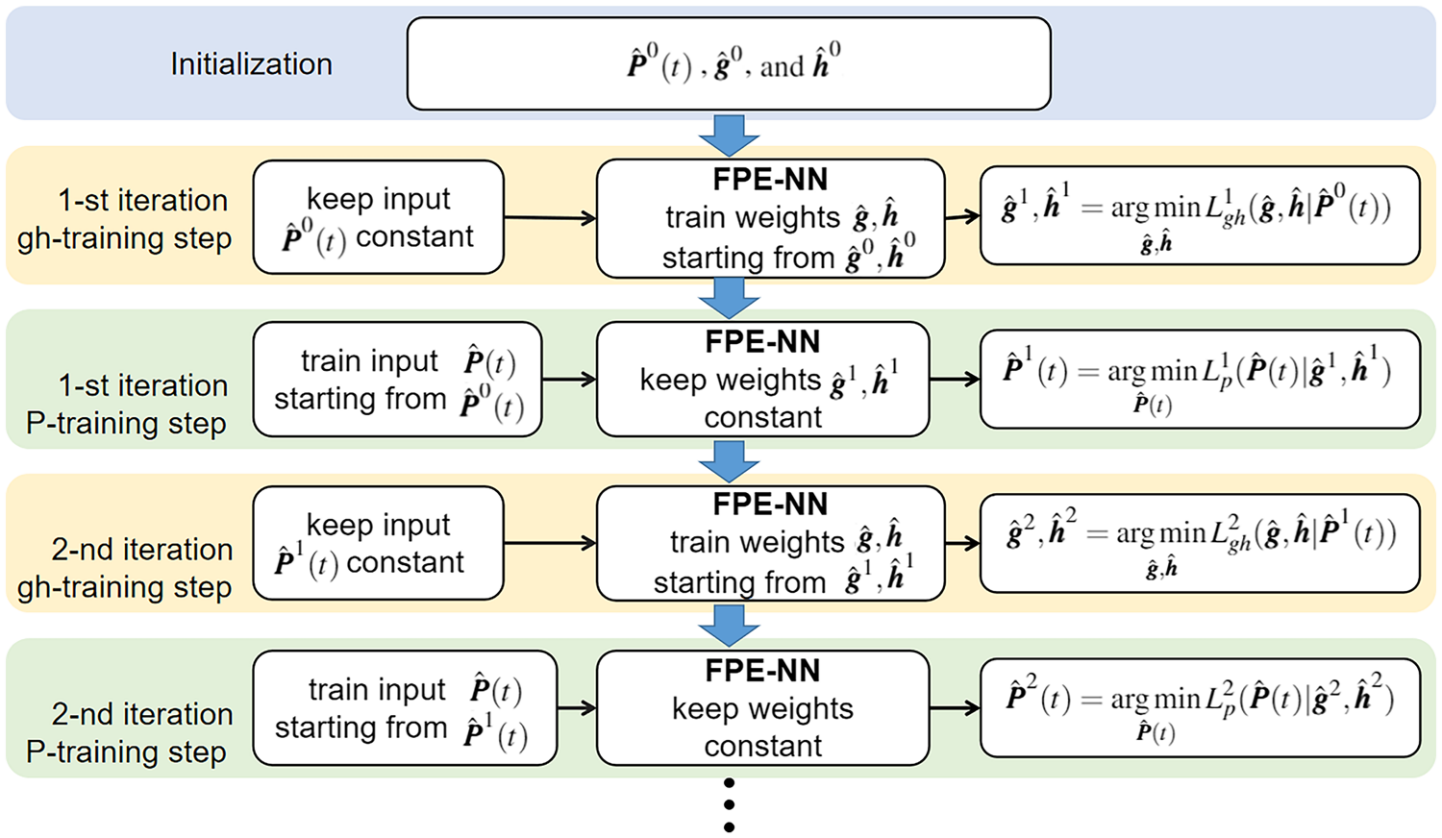
Flowchart of using FPE-NN to train the input ${\hat{\varvec{P}}}(t)$ and the weights ${\hat{\varvec{g}}}$, ${\hat{\varvec{h}}}$ in an alternating way. Each iteration consists of two steps, first the gh-training step then the P-training step. The counting of iteration starts from 1, as ${\hat{\varvec{P}}}^0(t)$, ${\hat{\varvec{g}}}^0$ and ${\hat{\varvec{h}}}^0$ are the initialized values. In the *k*-th iteration, the updated input and weights in the previous iteration are ${\hat{\varvec{P}}}^{k-1}(t)$, ${\hat{\varvec{g}}}^{k-1}$, and ${\hat{\varvec{h}}}^{k-1}$, respectively. We first keep ${\hat{\varvec{P}}}^{k-1}(t)$ constant to improve the accuracy of the weights in the gh-training step, and save the trained weights as ${\hat{\varvec{g}}}^k$ and ${\hat{\varvec{h}}}^k$. Then we keep ${\hat{\varvec{g}}}^k$ and ${\hat{\varvec{h}}}^k$ constant to improve the accuracy of the input in the P-training step, and save the trained input as ${\hat{\varvec{P}}}^k(t)$. Subsequently, ${\hat{\varvec{P}}}^k(t)$, ${\hat{\varvec{g}}}^k$, and ${\hat{\varvec{h}}}^k$ are used for the next iteration.

Generally, FPE-NN takes ${\varvec{P}}(t)$ at a time point as the input, ${\varvec{g}}$ and ${\varvec{h}}$ as trainable weights, to predict ${\varvec{P}}(t)$ at neighboring time points. The input and the weights are trained in an alternating way. To distinguish them from the true values, the training input and weights are denoted as ${\hat{\varvec{P}}}(t)$, ${\hat{\varvec{g}}}$, and ${\hat{\varvec{h}}}$, respectively. As shown in Fig. 1, each iteration of the training consists of two steps. The first step is the gh-training step, where we keep the updated ${\hat{\varvec{P}}}(t)$ constant to train ${\hat{\varvec{g}}}$ and ${\hat{\varvec{h}}}$. The second step is the P-training step, where we keep the updated ${\hat{\varvec{g}}}$ and ${\hat{\varvec{h}}}$ constant to train ${\hat{\varvec{P}}}(t)$. The alternating training process is repeated until the stopping criterion is met. The reason that the input and weights are not trained simultaneously is that we have to use different ways to feed in the data samples when training them. Specifically, ${\hat{\varvec{g}}}$ and ${\hat{\varvec{h}}}$ are time independent so the samples can be fed in batches, whereas ${\hat{\varvec{P}}}(t)$ is time dependent hence the samples have to be fed one-by-one.

### Architecture of FPE-NN

**Figure 2:**
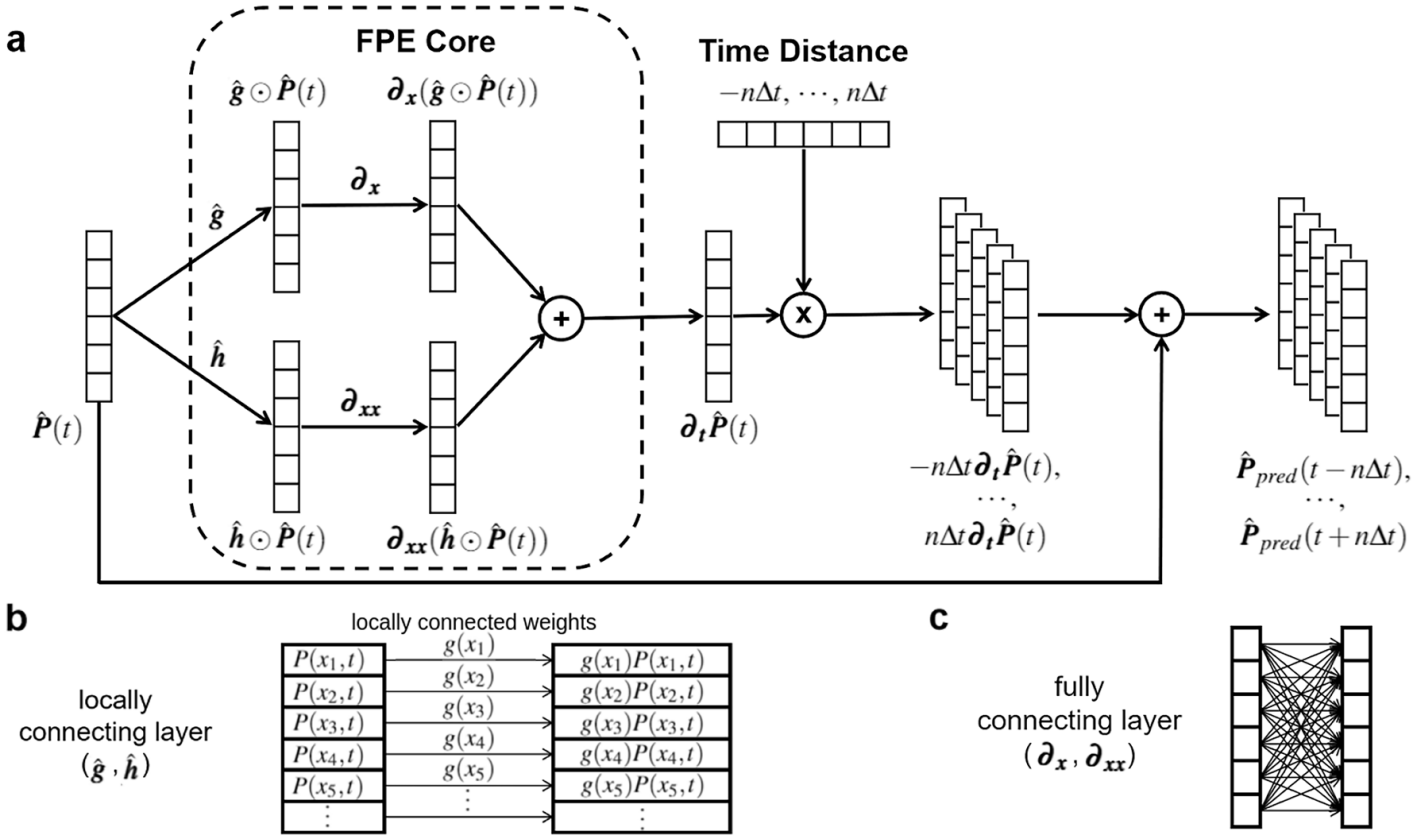
FPE-NN architecture. (**a**) The whole architecture of Fokker–Planck Equation Neural Network (FPE-NN). The time distance set used for training FPE-NN is a vector $(-n\Delta t, \ldots , n\Delta t)$, but it changes when we use FPE-NN to predict future distributions. The details are provided in the corresponding section later. (**b**) The architecture of locally connecting layers. (**c**) The architecture of fully connecting layers.

Here we introduce our FPE-based neural network model (FPE-NN). It consists of two major parts and each part consists of several layers (Fig. 2a). The central part, named FPE Core, is designed based on FPE which takes the input ${\hat{\varvec{P}}}(t)$ at a certain time point *t* to calculate the corresponding derivative ${\varvec{\partial }}_{\varvec{t}} {\hat{\varvec{P}}}(t)$ with weights ${\hat{\varvec{g}}}$ and ${\hat{\varvec{h}}}$. The remaining part is designed based on the Euler method which uses the input ${\hat{\varvec{P}}}(t)$ and the derived ${\varvec{\partial }}_{\varvec{t}} {\hat{\varvec{P}}}(t)$ to predict the corresponding distributions at multiple neighboring time points. Please take note that we are not using the neural network to approximate FPE or the Euler method, instead, we use the neural network to carry out the computation steps in FPE and the Euler method.

The network architecture of FPE Core is designed based on the discrete form of FPE over variable *x* for fixed-time *t*:

2 $$\begin{aligned} {\varvec{\partial }}_{\varvec{t}} {\varvec{P}}(t) = {\varvec{\partial }}_{\varvec{x}}({\varvec{g}} \odot {\varvec{P}}(t)) + \varvec{\partial }_{{{\varvec{x}}}{{\varvec{x}}}}({\varvec{h}} \odot {\varvec{P}}(t)) \end{aligned}$$

where $\odot$ is the element-wise multiplication; ${\varvec{\partial }}_{\varvec{x}}$ and $\varvec{\partial }_{{{\varvec{x}}}{{\varvec{x}}}}$ are matrices to compute derivatives following a new and accurate finite difference method^21^. The element-wise multiplication of two vectors is implemented by locally connecting two layers (Fig. 2b) and the multiplication of a vector by a matrix is achieved by fully connecting two layers (Fig. 2c). The weights of the locally connecting layers are the FPE terms ${\hat{\varvec{g}}}$ and ${\hat{\varvec{h}}}$, which are trainable. The weights of the fully connecting layers are non-trainable and have fixed values that perform derivative calculations ${\varvec{\partial }}_{\varvec{x}}$ and $\varvec{\partial }_{{{\varvec{x}}}{{\varvec{x}}}}$ (details are provided in the Supplementary). There are no biases or activation functions in these two kinds of connecting layers. Overall, the FPE Core computes the corresponding ${\varvec{\partial }}_{\varvec{t}} {\hat{\varvec{P}}}(t)$ of the input ${\hat{\varvec{P}}}(t)$ at a certain time point *t* based on the given weights ${\hat{\varvec{g}}}$ and ${\hat{\varvec{h}}}$.

In the following part, we use the input ${\hat{\varvec{P}}}(t)$ and the derived ${\varvec{\partial }}_{\varvec{t}} {\hat{\varvec{P}}}(t)$ from FPE Core to predict the distribution at time point $t + i\Delta t$ using the Euler method, where $i\in \{-n, \ldots , n\}$ and *n* is a hyperparameter to be tuned case-by-case. $\Delta t$ is the time gap between adjacent time points. As shown in Fig. 2a, a few layers are added to perform the following calculation:

3 $$\begin{aligned} {\hat{\varvec{P}}}_{pred}(t+i\Delta t)&= {\hat{\varvec{P}}}(t) + i\Delta t {\varvec{\partial }}_{\varvec{t}} {\hat{\varvec{P}}}(t) \end{aligned}$$

where ${\hat{\varvec{P}}}_{pred}(t + i\Delta t)$ is the predicted distribution at time point $t + i\Delta t$. There is no need to add an extra constraint to ensure that the integral of ${\hat{\varvec{P}}}_{pred}(t + i\Delta t)$ equals to 1, because the integral of a pdf is invariant when it evolves according to FPE (Proof is provided in the Supplementary). Furthermore, the integrals of the target distributions all equal to 1, and ${\hat{\varvec{P}}}_{pred}(t + i\Delta t)$ is forced to be very close to the target distributions by the loss functions. The details about the target distributions and the loss functions are provided later.

In summary, the inputs for the whole network FPE-NN is ${\hat{\varvec{P}}}(t)$, the trainable weights are ${\hat{\varvec{g}}}$ and ${\hat{\varvec{h}}}$, and the output is the predicted distributions ${\hat{\varvec{P}}}_{pred}(t + i\Delta t)$, $i\in \{-n, \ldots , n\}$. In order to generate a loss function for training, ${\hat{\varvec{P}}}_{pred}$ has to be compared with target distributions. However, as the true pdf ${\varvec{P}}_{clean}$ is unknown, we can only compare ${\hat{\varvec{P}}}_{pred}$ with some selected noisy target distributions. We will describe how we formulate the loss functions using the noisy target distributions when the details of the alternating training steps are provided. Furthermore, in order to alleviate the noise effect of the target distributions, FPE-NN is designed to make predictions at $2n + 1$ time points and usually $n > 1$. Detailed explanations are provided in the Supplementary.

### Initialization

The initialized input ${\hat{\varvec{P}}}(t)$, denoted as ${\hat{\varvec{P}}}^0(t)$, is derived by smoothing ${\varvec{P}}_{noisy}(t)$ with the Savitzky-Golay (SG) filter^23^, which helps to partially remove the noise. The initialized weights, denoted as ${\hat{\varvec{g}}}^0$ and ${\hat{\varvec{h}}}^0$, are calculated based on ${\hat{\varvec{P}}}^0(t)$ using a linear least square method (details are provided in the Supplementary). In the following two subsections, we will explain how to train and update the input and weights separately in the *k*-th iteration.

### The gh-training step in the *k*-th iteration

In the beginning of the *k*-th training iteration ($k=1,2, \dots$), ${\hat{\varvec{P}}}(t)$, ${\hat{\varvec{g}}}$ and ${\hat{\varvec{h}}}$ have been updated in the previous iteration. The updated values are denoted as ${\hat{\varvec{P}}}^{k-1}(t)$, ${\hat{\varvec{g}}}^{k-1}$ and ${\hat{\varvec{h}}}^{k-1}$, respectively. For the first iteration ($k=1$), we use the initialized input ${\hat{\varvec{P}}}^0(t)$ and weights ${\hat{\varvec{g}}}^0$ and ${\hat{\varvec{h}}}^0$. Then we first run the gh-training step to train ${\hat{\varvec{g}}}$ and ${\hat{\varvec{h}}}$ (yellow blocks in Fig. 1). Specifically, the input of FPE-NN would be ${\hat{\varvec{P}}}^{k-1}(t)$ which is kept constant in this step, and the weights are ${\hat{\varvec{g}}}$ and ${\hat{\varvec{h}}}$ which are trainable with initial values ${\hat{\varvec{g}}}^{k-1}$ and ${\hat{\varvec{h}}}^{k-1}$, respectively. The output ${\hat{\varvec{P}}}_{pred}$ is compared with ${\hat{\varvec{P}}}^{k-1}$, hence one data sample consists of a single distribution ${\hat{\varvec{P}}}^{k-1}(t)$ as input and multiple distributions at neighboring time points ${\hat{\varvec{P}}}^{k-1}(t + i\Delta t)$ as target output, where $i\in \{-n, \ldots , n\}$. The loss function is:

4 $$\begin{aligned} L_{gh}^k({\hat{\varvec{g}}},{\hat{\varvec{h}}}|{\hat{\varvec{P}}}^{k-1}(t)) &=\sum _t\sum _{i=-n}^{n}\left \|{ {\hat{\varvec{P}}}_{pred}(t+i\Delta t) - {\hat{\varvec{P}}}^{k-1}(t+i\Delta t) }\right\| _2^2 \\ &=\sum _t\sum _{i=-n}^{n}\left\|{ {\hat{\varvec{P}}}^{k-1}(t) + i\Delta t ({{\partial }}_{\varvec{x}}({\hat{\varvec{g}} {\hat{P}}}^{k-1}(t)) + {\partial}_{{{\varvec{x}}}{{\varvec{x}}}}({\hat{\varvec{h}}{\hat{P}}}^{k-1}(t)))-{\hat{\varvec{P}}}^{k-1}(t+i\Delta t)}\right\| ^2_2 \end{aligned}$$

where the superscript *k* indicates the k-th iteration. Because ${\hat{\varvec{g}}}$ and ${\hat{\varvec{h}}}$ are time-independent and shared by all the data samples, we can train FPE-NN in batch training by randomly picking a certain number of samples, which is the first summation in Eq. (4). The interpretation of $L_{gh}^k$ is that we assume ${\hat{\varvec{P}}}^{k-1}(t)$ satisfies FPE and search for the optimal terms. The optimized ${\hat{\varvec{g}}}$ and ${\hat{\varvec{h}}}$ are saved as the updated weights of the *k*-th iteration.

5 $$\begin{aligned} {\hat{\varvec{g}}}^k, {\hat{\varvec{h}}}^k = \mathop {\text {arg min}}\limits _{{\hat{\varvec{g}}}, {\hat{\varvec{h}}}} L_{gh}^k({\hat{\varvec{g}}},{\hat{\varvec{h}}}| {\hat{\varvec{P}}}^{k-1}(t)) \end{aligned}$$

where ${\hat{\varvec{P}}}^{k-1}(t)$ is kept constant.

### The P-training step in the *k*-th iteration

After the gh-training step of the *k*-th iteration, the latest updated input and weights are ${\hat{\varvec{P}}}^{k-1}(t)$, ${\hat{\varvec{g}}}^k$ and ${\hat{\varvec{h}}}^k$, respectively. Now we run the P-training step to train ${\hat{\varvec{P}}}(t)$ (green blocks in Fig. 1). Specifically, FPE-NN would have a trainable input ${\hat{\varvec{P}}}(t)$ with initial value ${\hat{\varvec{P}}}^{k-1}(t)$, and the weights ${\hat{\varvec{g}}}^k$ and ${\hat{\varvec{h}}}^k$ which are kept constant in this step. The output ${\hat{\varvec{P}}}_{pred}$ is compared with ${\hat{\varvec{P}}}^0$ which is the smoothed ${\varvec{P}}_{noisy}$. Therefore, one data sample has a single distribution ${\hat{\varvec{P}}}^{k-1}(t)$ as input and multiple distributions ${\hat{\varvec{P}}}^0(t + i\Delta t)$ as target output, where $i\in \{-n, \ldots , n\}$. The corresponding loss function is:

6 $$\begin{aligned} L_{p}^k({\hat{\varvec{P}}}(t)|{\hat{\varvec{g}}}^k, {\hat{\varvec{h}}}^k)&= \sum _{i=-n}^{n}\left\|{ {\hat{\varvec{P}}}_{pred}(t + i\Delta t) - {\hat{\varvec{P}}}^0(t + i\Delta t)} \right\|_{2}^{2} \\&= \sum _{i=-n}^{n}\left\|{{\hat{\varvec{P}}}(t) + i\Delta t ({{\partial }}_{\varvec{x}}({\hat{\varvec{g}}}^k {\hat{\varvec{P}}}(t)) + {\partial }_{{{\varvec{x}}}{{\varvec{x}}}}({\hat{\varvec{h}}}^k {\hat{\varvec{P}}}(t))) - {\hat{\varvec{P}}}^0(t + i\Delta t)}\right\|^{2}_{2} \end{aligned}$$

where the superscript *k* indicates the k-th iteration. Because the input ${\hat{\varvec{P}}}(t)$ is time-dependent and specific to each sample, we could only train the samples one-by-one. The interpretation of $L_{P}^k$ is that we search for the optimal ${\hat{\varvec{P}}}(t)$ which has the minimum difference to ${\hat{\varvec{P}}}^{0}(t)$, and naturally to ${\hat{\varvec{P}}}_{noisy}(t)$, when it evolves according to FPE with terms ${\hat{\varvec{g}}}^k$ and ${\hat{\varvec{h}}}^k$. The optimized ${\hat{\varvec{P}}}(t)$ is saved as the updated input of the *k*-th iteration:

7 $$\begin{aligned} {\hat{\varvec{P}}}^k(t) = \mathop {\text {arg min}}\limits _{{\hat{\varvec{P}}}(t)} L_p^k ({\hat{\varvec{P}}}(t)|{\hat{\varvec{g}}}^k, {\hat{\varvec{h}}}^k) \end{aligned}$$

where ${\hat{\varvec{g}}}^k$ and ${\hat{\varvec{h}}}^k$ are kept constant.

### Training Stopping Criterion

As described previously, the alternating FPE-NN training process uses the updated ${\hat{\varvec{P}}}(t)$ to improve the accuracy of ${\hat{\varvec{g}}}$ and ${\hat{\varvec{h}}}$, and uses the updated ${\hat{\varvec{g}}}$ and ${\hat{\varvec{h}}}$ to improve the accuracy of ${\hat{\varvec{P}}}(t)$. Eventually, FPE-NN aims to recover the true values of ${\hat{\varvec{P}}}(t)$, ${\hat{\varvec{g}}}$ and ${\hat{\varvec{h}}}$. However, because the true values are unknown, an indirect metric must be set to evaluate the training process and used as a criterion to stop the training iteration. In this study we use the sum of the two loss functions $L_{gh}+L_{P}$ as the stopping criterion, to stop the whole training process when it converges or fails to decrease within a certain number of successive iterations.

### Predict future distributions

Once the whole training process is finished, the well-trained weights ${\hat{\varvec{g}}}$ and ${\hat{\varvec{h}}}$ could be used to predict the future evolution of a different pdf, denoted as ${\varvec{P}}'_{noisy}(t)$, which satisfies the same FPE but is not used in FPE-NN training. Specifically, the task would be to use the distributions of ${\varvec{P}}'_{noisy}(t)$ at time points $\{t -r \Delta t, \ldots , t\}$ to predict the distributions at time points $\{t + \Delta t, \ldots , t + f \Delta t\}$, where *r* and *f* are user-defined parameters. In this study, we choose $r = n$ and $f = 5$.

The prediction process is similar to the P-training step which keeps the FPE-NN weights constant. First, the given distributions ${\varvec{P}}'_{noisy}(\cdot )$ are smoothed with the SG filter, and the smoothed distributions are denoted by ${\varvec{P}}''_{noisy}(\cdot )$. We then use ${\varvec{P}}''_{noisy}(\cdot )$ as the target output to train the input ${\hat{\varvec{P}}}(t)$ using FPE-NN. The corresponding time distance set is $\{-r \Delta t, \ldots , 0\}$ which is different from the one used in the FPE-training process (Fig. 1). Hence the corresponding loss function becomes:

8 $$\begin{aligned} L_{app}({\hat{\varvec{P}}}(t)|{\hat{\varvec{g}}}, {\hat{\varvec{h}}})= \sum_{i=-r}^{0}\left\|{ {\hat{\varvec{P}}}(t) + i\Delta t ({{\partial }}_{\varvec{x}}({\hat{\varvec{g}}} {\hat{\varvec{P}}}(t)) + {\partial}_{{{\varvec{x}}}{{\varvec{x}}}}({\hat{\varvec{h}}}{\hat{\varvec{P}}}(t))) - {\varvec{P}}^{\prime\prime}_{noisy}(t + i\Delta t)}\right\|^{2}_{2}\end{aligned}$$

where ${\hat{\varvec{g}}}$ and ${\hat{\varvec{h}}}$ are well-trained weights and kept constant. By minimizing the loss value we derive the optimal ${\hat{\varvec{P}}}(t)$:

9 $$\begin{aligned} {\hat{\varvec{P}}}(t) = \mathop {\text {arg min}}\limits _{{\hat{\varvec{P}}}(t)} L_{app} ({\hat{\varvec{P}}}(t)|{\hat{\varvec{g}}}, {\hat{\varvec{h}}}) \end{aligned}$$

Finally, the optimized ${\hat{\varvec{P}}}(t)$ is fed into FPE-NN again with another time distance set $\{\Delta t, \ldots , f \Delta t\}$, to predict distributions at time points $\{t + \Delta t, \ldots , t + f \Delta t\}$:

10 $$\begin{aligned} {\hat{\varvec{P}}}_{pred}(t+i\Delta t) = {\hat{\varvec{P}}}(t) + i\Delta t ({\varvec{\partial }}_{\varvec{x}}({\hat{\varvec{g}}} {\hat{\varvec{P}}}(t)) + \varvec{\partial }_{{{\varvec{x}}}{{\varvec{x}}}}({\hat{\varvec{h}}}{\hat{\varvec{P}}}(t))) \end{aligned}$$

where $$i \in \{1, \ldots , f \}$$.

## Numerical studies

### Evaluation metrics

The training process can be evaluated with up to six metrics. The first two metrics are the loss values, which can be used to quantify the training result based on the observation ${\varvec{P}}_{noisy}(t)$. In particular $L_{gh}$ measures how well $\hat{{\varvec{P}}}(t)$ satisfies FPE, while $L_{P}$ measures how close $\hat{{\varvec{P}}}(t)$ is to the measured pdf ${\varvec{P}}_{noisy}(t)$.

In the cases of validating our methods with simulated data where the corresponding true values are known, three more metrics can be used to quantify the normalized errors of $\hat{{\varvec{P}}}(t)$, ${\hat{\varvec{g}}}$ and ${\hat{\varvec{h}}}$:

11 $$\begin{aligned} E_{P} = \frac{\parallel {\hat{\varvec{P}}}(t) - {\varvec{P}}_{clean}(t)\parallel _2}{\parallel {\varvec{P}}_{clean}(t) \parallel _2}, \qquad E_{g} = \frac{\parallel {\hat{\varvec{g}}} - {\varvec{g}}\parallel _2}{\parallel {\varvec{g}} \parallel _2}, \qquad E_{h} = \frac{\parallel {\hat{\varvec{h}}} - {\varvec{h}}\parallel _2}{\parallel {\varvec{h}} \parallel _2} \end{aligned}$$

where ${\varvec{P}}(t)$, ${\varvec{g}}$ and ${\varvec{h}}$ are the true values. According to the form of the loss function $L_{gh}$ (Eq. (4)), the gradients of ${\hat{\varvec{g}}}$ and ${\hat{\varvec{h}}}$ both are proportional to the corresponding ${\hat{\varvec{P}}}(t)$ at the same point of *x*. Usually the values of ${\hat{\varvec{P}}}(t)$ at the boundary points of *x* are very small, hence the corresponding ${\hat{\varvec{g}}}$ and ${\hat{\varvec{h}}}$ at these points are difficult to be well trained in backpropagation. On the other hand, the boundary points are less important due to their small ${\varvec{P}}(t)$ values. Hence, we also measure the error of ${\hat{\varvec{g}}}$ and ${\hat{\varvec{h}}}$ excluding the boundary points, denoted as $\tilde{E}_g$ and $\tilde{E}_h$, respectively. Please take note that the boundary area is not excluded during the training process, they are ignored only when calculating $\tilde{E}_g$ and $\tilde{E}_h$. The boundary area is determined by using the student T-test^24^ at each point of *x*. The *x* points whose density values within all the training data are statistically lower than $0.01 \times max({\varvec{P}}_{noisy}(t))$ are classified to the boundary area.

The last metric is the ability of using the trained ${\hat{\varvec{g}}}$ and ${\hat{\varvec{h}}}$ to predict future distributions, the detailed process of which has been described previously. The normalized error of the predicted future distribution is denoted as $E_{test}$:

12 $$\begin{aligned} E_{test} = \frac{\parallel {\hat{\varvec{P}}}_{pred}(t+i \Delta t) - {\varvec{P}}_{target}(t + i \Delta t)\parallel _2}{\parallel {\varvec{P}}_{target}(t + i \Delta t) \parallel _2} \end{aligned}$$

where $i \in \{1, \ldots , f\}$ indicates the number of time gaps ahead of the last known distribution, ${\hat{\varvec{P}}}_{pred}(t + i\Delta t)$ is the predict distribution provided in Eq. (10), and ${\varvec{P}}_{target}(t + i\Delta t)$ is the ground truth.

### Simulated data

We validate our method with simulated data. First, We solve the forward problem of FPE with chosen FPE terms and initial conditions, to generate several distribution sequences at multiple time points. Then, we assume the FPE terms are unknown, and we train FPE-NN to solve the inverse problem of FPE which is to find the FPE terms based on the generated distribution sequences.

The simulated data is generated based on three selected FPE examples which have been used to model real-world phenomena in different areas. Another FPE example, the Brownian motion in a periodical potential^25^, is also used to validate our method and the result is provided in the Supplementary. For each FPE example, we generate 120 distribution sequences and each sequence consists of distributions at 50 time points with a uniform time gap $\Delta t$. 100 distribution sequences are used to train FPE-NN and the remaining 20 sequences are used to test the ability of predicting future distributions. We also tested training with less or more than 100 distribution sequences, and the result is provided in the Supplementary.

#### Example 1: Heat flux (OU process)

Our first FPE example comes from meteorology, where Lin and Koshyk^26^ used it in climate dynamics to describe how the heat flux at the sea surface evolves. The observable *x* is the deviation of the heat flux from its steady state in units of watts per square meter. Each unit of time *t* corresponds to 55 days. The FPE form is:

13 $$\begin{aligned} \frac{\partial P(x,t)}{\partial t}= \theta \frac{\partial }{\partial x}(x P(x,t)) + D\frac{\partial ^2 P(x,t)}{\partial x^2} \end{aligned}$$

The process described by Eq. (13) is called the Ornstein-Uhlenbeck (OU) process^17^, where *D* and $\theta$ are the diffusion and drift coefficients respectively. It was originally used in the Brownian motion of molecular dynamics and has since been applied widely in various fields^27–29^. The forward problem of Eq. (13) has an analytical solution which is a time-varying Gaussian distribution:

14 $$\begin{aligned} P(x,t) = \sqrt{\frac{1}{2\pi \sigma ^2(t)}}exp\bigg ({-\frac{(x-\mu (t))^2}{2\sigma ^2(t)}}\bigg ) \end{aligned}$$

where the mean $\mu$ and deviation $\sigma$ are:

15 $$\begin{aligned} \sigma (t) = D(1-e^{-2\theta t} )/ \theta , \qquad \mu (t) = x_0 e^{-\theta t}. \end{aligned}$$

where $x_0$ is the origin state of *x* when $t=0$. We set $D=0.0013$ and $\theta =2.86$, which are the empirical parameters from the sea measurements^30^. *x* is discretized into 110 bins with support $[-0.01, 0.1]$. The initial conditions are uniformly sampled from $x_0 \in [0.04, 0.08]$ and $t_{init} \in [0.03, 0.05]$. We obtain simulations with a random pair of $(x_0, t_{init})$ for each distribution sequence. We adjust the time gap $\Delta t$ to be 0.001 in order to maximize the distribution differences at different time points while keeping *P*(*x*, *t*) values at boundary points close to zero. The time gap of the next two examples are also tuned based on the same criteria.

#### Example 2: DNA bubble (Bessel process)

The next FPE example is from the biology area, where FPE has been used to model the dynamics of DNA bubbles formed due to thermal fluctuations^16^. The observable *x* is the bubble length in units of base pair and the time variable *t* is in units of microsecond. The corresponding pdf satisfies:

16 $$\begin{aligned} \frac{\partial P(x,t)}{\partial t} = \frac{\partial }{\partial x}\left( \left( \frac{\mu }{x}-\epsilon \right) P(x,t)\right) + \frac{1}{2}\frac{\partial ^2 P(x,t)}{\partial x^2} \end{aligned}$$

Equation (16) describes a generalized Bessel process which also has many other applications^31^. In the DNA bubble study, $\mu$ is estimated as 1 and $\epsilon$ is approximated as $2(T/T_m - 1)$, where *T* is the environment temperature and $T_m$ is the melting temperature which is determined by the DNA sequence. In this work, we set $\mu = 1$ to follow the DNA bubble study and arbitrarily choose $\epsilon =0.2$. Equation (16) has a very complicated analytical solution^31^. Hence for convenience, we generate data by solving the forward problem with the 4th-order Runge–Kutta method for temporal discretization. *x* is discretized into 100 bins with support [0.1, 1.1]. The initial condition is a normal distribution whose mean $\mu$ and deviation $\sigma$ are randomly sampled from [0.4, 0.8] and [0.05, 0.1] respectively. We adjust the time gap $\Delta t$ to be 0.001, based on the same criteria as mentioned before.

#### Example 3: Agent’s wealth

The final FPE example comes from economics, where Cordier et al.^7^ uses FPE to describe the wealth distribution of agents. The agent is a terminology in economics referring to an individual, company, or organization who has an influence on the economy through producing, buying, or selling. The observable *x* is the agent’s wealth and its pdf satisfies:

17 $$\begin{aligned} \frac{\partial P(x,t)}{\partial t} = \frac{\lambda }{2} \frac{\partial ^2}{\partial x^2}(x^2 P(x,t)) + \frac{\partial }{\partial x}((x - m)P(x,t)) \end{aligned}$$

Equation (17) has no analytical solution and we solve the forward problem by using the 4th-order Runge–Kutta method. We set $\lambda = 0.4$ and $m = 1$ following Cordier’s report^7^. *x* is discretized into 100 bins with support [0, 1.0]. Similar to the DNA bubble example, the initial condition is a normal distribution whose $\mu$ and $\sigma$ are randomly sampled from [0.3, 0.7] and [0.05, 0.1], respectively. The time gap $\Delta t$ is adjusted to be $5\text {e-}4$, based on the same criteria mentioned in the heat flux example.

#### Noise addition

After we generate the distribution sequences (denoted as $P_{clean}$) by solving the forward problem of FPE as described previously, noise is added to the distributions to model real-world scenarios:

18 $$\begin{aligned} P_{noisy}(x,t) = (1 + W \xi )P_{clean}(x,t) \end{aligned}$$

where $\xi$ is a random number sampled from the standard normal distribution *N*(0, 1), and *W* is a constant which is adjusted to ensure that the corresponding $E_P$ of $P_{noisy}(x,t)$ is approximately 0.01. Simulated data with higher corresponding $E_P$ is also tested using our method, and the results are provided in the Supplementary.

**Figure 3:**
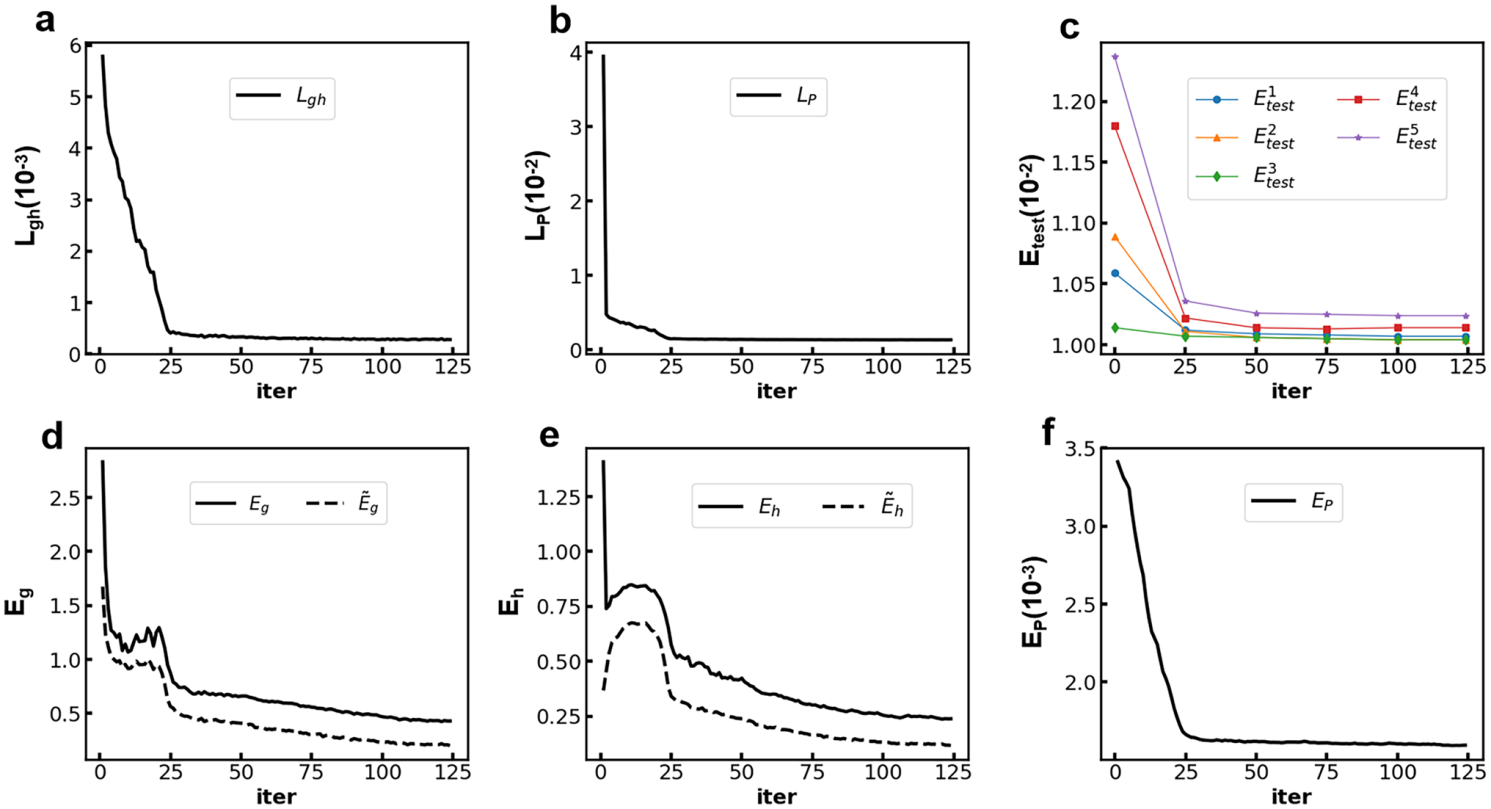
Curves of a typical training process with the data of the agents’ wealth. The training is stopped when the sum of $L_{gh}$ and $L_{P}$ fails to decrease within 20 successive iterations. (**a**) $L_{gh}$. (**b**) $L_{P}$. (**c**) $E_{test}$. (d) $E_{g}$ (solid line) and $\tilde{E}_g$ (dash line). (**e**) $E_{h}$ (solid line) and $\tilde{E}_h$ (dash line). (**f**) $E_{P}$.

### Results

As described previously, we generate the simulated data by solving the forward problem of FPE with given FPE terms and initial conditions. After that, we assume the FPE terms are completely unknown, and train FPE-NN to solve the inverse problem which is to find the FPE terms based on the simulated data. A typical training process with the data from the agents’ wealth example is shown in Fig. 3. $L_{P}$ decreases rapidly in the first few iterations while $L_{gh}$ decreases at a slower rate. Both of their curves become almost flat after the 25-th iteration and decrease very slowly. The curve of $E_P$ generally coincides with that of $L_{P}$ with a turning point at the 25-th iteration. In contrast, $E_g$ and $E_h$ are continuously decreasing during the iterations. The result demonstrates that with the alternating training process, the input and weights help each other gradually recover the true values over the iterations. We also found that further improvements on the accuracy of ${\hat{\varvec{g}}}$ and ${\hat{\varvec{h}}}$, after reaching a certain point, have minor effect on the accuracy improvement of ${\hat{\varvec{P}}}(t)$. On the other hand, ${\hat{\varvec{g}}}$ and ${\hat{\varvec{h}}}$ are more sensitive to the minor improvements in ${\hat{\varvec{P}}}(t)$. We also test $E_{test}$ at several selected iterations as shown in Fig. 3c. The result suggests that a larger time gap is more sensitive to the accuracy of ${\hat{\varvec{g}}}$ and ${\hat{\varvec{h}}}$. However, further improvement in ${\hat{\varvec{g}}}$ and ${\hat{\varvec{h}}}$ after a certain point has little effect on future distribution prediction. A typical plot of the predicted distributions in future time is provided in Supplementary. Overall, the learning curves demonstrate our self-supervised method can train ${\hat{\varvec{P}}}(t)$, ${\hat{\varvec{g}}}$ and ${\hat{\varvec{h}}}$ in an alternating way and let them gradually recover the true values.

**Figure 4:**
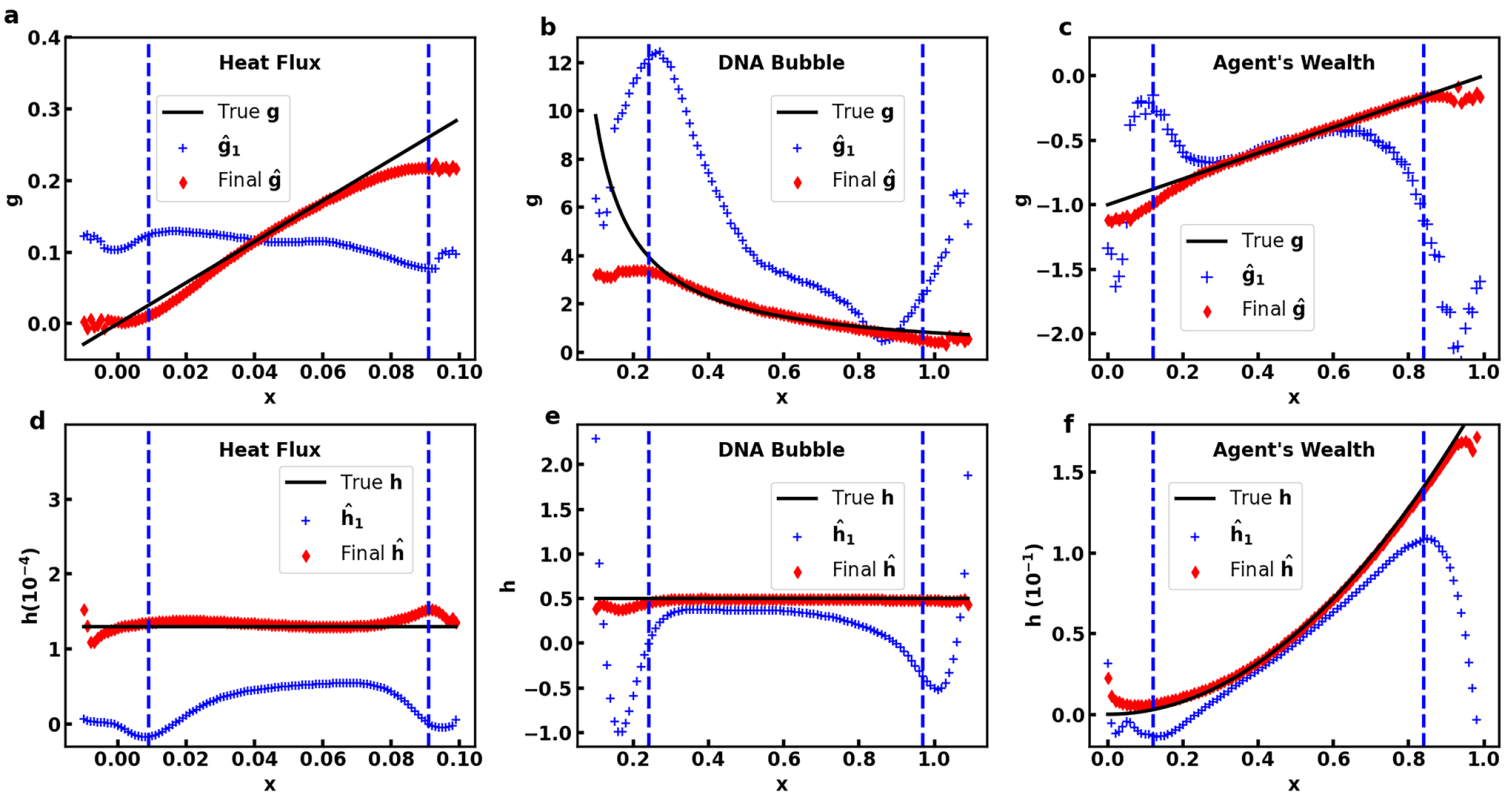
Comparison of the true ${\varvec{g}}$ and ${\varvec{h}}$ (black line) and the trained weights ${\hat{\varvec{g}}}$ and ${\hat{\varvec{h}}}$ from the first training iteration (blue cross) and the final iteration (red diamond) in heat flux (**a**, **d**), DNA bubble (**b**, **e**), and agent’s wealth (**c**, **f**) examples. The boundary area is indicated by the vertical blue dash line. ${\hat{\varvec{g}}}^1$ and ${\hat{\varvec{h}}}^1$ are the trained weights in the first iteration. They are the optimal FPE terms can be found based on the smoothed ${\varvec{P}}_{noisy}(t)$, ${\hat{\varvec{P}}}^0(t)$. The figure shows that ${\hat{\varvec{g}}}^1$ and ${\hat{\varvec{h}}}^1$ are significantly different from their true values, suggesting that merely smoothing ${\varvec{P}}_{noisy}(t)$ is insufficient. To further reduce the noise by training ${\hat{\varvec{P}}}(t)$ with FPE-NN is essential for the ${\hat{\varvec{g}}}$ and ${\hat{\varvec{h}}}$ optimisation.

**Table 1:** Summary of the evaluation metric values of the simulated data from three FPE examples. The number of the training distribution sequences is fixed as 100. *n* is the output time point range used in Eqs. (4) and (6).

Example	*n*	$L_{gh}/(2n+1)$	$L_{P}/(2n+1)$	$E_g(\tilde{E}_g)$	$E_h(\tilde{E}_h)$	$E_P$	$E^1_{test}$	$E^5_{test}$
Flux	2	1.07e−4	1.27e−4	0.138 (0.076)	0.228 (0.065)	0.0028	0.010 ± 0.001	0.011 ± 0.002
Flux	4	1.73e−4	2.15e−4	0.134 (0.085)	0.148 (0.072)	0.0028	0.010 ± 0.001	0.011 ± 0.002
Flux	6	2.22e−4	3.67e−4	0.154 (0.084)	0.158 (0.093)	0.0033	0.010 ± 0.001	0.011 ± 0.002
Flux	8	4.42e−4	4.93e−4	0.292 (0.104)	0.212 (0.099)	0.0037	0.010 ± 0.001	0.011 ± 0.002
Bubble	2	1.20e−4	3.91e−4	0.377 (0.148)	0.046 (0.041)	0.0021	0.011 ± 0.002	0.011 ± 0.002
Bubble	4	1.51e−4	6.30e−4	0.407 (0.162)	0.036 (0.030)	0.0017	0.011 ± 0.002	0.011 ± 0.002
Bubble	6	2.23e−4	7.85e−4	0.415 (0.184)	0.033 (0.022)	0.0015	0.011 ± 0.002	0.011 ± 0.002
Bubble	8	3.84e−4	9.28e−4	0.390 (0.157)	0.043 (0.028)	0.0014	0.011 ± 0.002	0.011 ± 0.002
Wealth	2	2.52e−4	8.92e−4	0.083 (0.040)	0.049 (0.026)	0.0023	0.010 ± 0.001	0.010 ± 0.002
Wealth	4	2.33e−4	1.29e−3	0.110 (0.045)	0.061 (0.020)	0.0018	0.010 ± 0.001	0.010 ± 0.002
Wealth	6	2.69e−4	1.35e−3	0.417 (0.200)	0.233 (0.116)	0.0016	0.010 ± 0.001	0.010 ± 0.002
Wealth	8	3.11e−4	1.66e−3	0.359 (0.191)	0.281 (0.138)	0.0016	0.010 ± 0.001	0.010 ± 0.002

The training results of the three different FPE examples are summarized in Table 1. Generally, ${\hat{\varvec{P}}(t)}$, ${\hat{\varvec{g}}}$ and ${\hat{\varvec{h}}}$ have been well trained using FPE-NN in all the three simulated datasets. The noise of the trained ${\hat{\varvec{P}}}(t)$ is significantly suppressed, as the final noise ratio $E_P$ could reach to less than 0.002. In contrast, the corresponding $E_P$ for ${\varvec{P}}_{noisy}(t)$ is around 0.01. The trained ${\hat{\varvec{g}}}$ and ${\hat{\varvec{h}}}$ also fit well with their true values. Figure 4 shows the plots of the final values of ${\hat{\varvec{g}}}$ and ${\hat{\varvec{h}}}$ (red diamond), excluding the boundary points, are quite close to the true values (black line). In contrast, if we do not train the input but just smooth ${\varvec{P}}_{noisy}(t)$ with SG-filter, the optimized weights ${\hat{\varvec{g}}}^1$ and ${\hat{\varvec{h}}}^1$ (blue cross) have much higher error. Hence, further reduction of the noise in ${\varvec{P}}_{noisy}(t)$ is essential to improving the accuracy of trained ${\hat{\varvec{g}}}$ and ${\hat{\varvec{h}}}$.

We also examined how the training result is affected by the number of output distributions, which is the hyperparameter *n* in Eqs. (4) and (6). As demonstrated in the Supplementary, $n>1$ can help to suppress the noise effect of ${\hat{\varvec{P}}}(t)$. However, it is not always better to have more neighboring time points, because the local truncation error from the Euler method may become too large to be ignored. Therefore *n* is a hyperparameter which needs to be tuned case-by-case. In this study, we tested four different values of *n* for each FPE example. As shown in Table 1, $L_{gh}$ and $L_{P}$ always increase when *n* increases, which is because of the larger truncation error caused by the larger time distance. However, the errors of ${\hat{\varvec{P}}}(t)$, ${\hat{\varvec{g}}}$ and ${\hat{\varvec{h}}}$ are not monotonically increasing or decreasing. Different datasets have different best *n* within the four options.

The effect of the training data size is also studied, by training FPE-NN with 200, 75, or 50 distribution sequences. The detailed result is provided in the Supplementary. Generally, using less data (75, or 50 sequences) causes the calculated ${\hat{\mathbf {g}}}$ and ${\hat{\mathbf {h}}}$ to be less accurate. More distribution sequences could provide more information about how the PDF evolves, enabling better fit of *g*(*x*) and *h*(*x*). On the other hand, the accuracy of the training result can be further improved by using more training data (200 sequences), but in real applications it could be too expensive to acquire extra distributions. Hence, the result shows that 100 distribution sequences is an appropriate data size to achieve accurate enough training results.

## Discussion

In this study, we proposed a new method to find the FPE terms based on the measured noisy pdfs, without any prior knowledge except assuming the pdfs satisfy FPE. Such an inverse problem of FPE could help us to study the stochastic processes in a more efficient way. We designed our network FPE-NN based on FPE and the Euler method. The input of FPE-NN is the distribution at a time point, the trainable weights are ${\hat{\varvec{g}}}$ and ${\hat{\varvec{h}}}$ representing the FPE terms, and the output is the distributions at several neighboring time points. In an alternating way, we use FPE-NN to train the input and the weights separately, forcing them to recover their true values. By training the input we successfully reduced the noise in the measured pdfs, which is a key factor for finding the accurate FPE terms. We validated our method with simulated data from three typical FPE examples. The result shows that the final trained input and weights all can agree well with the true values.

Although we are using a uniform grid to generate the simulated data in this paper, FPE-NN also can be used with minor changes in cases where the spatial gap $\Delta x$ and the time gap $\Delta t$ are not uniform. As shown in the Supplementary, the matrices which perform the derivative calculations of ${\varvec{\partial }}_{\varvec{x}}$ and $\varvec{\partial }_{{{\varvec{x}}}{{\varvec{x}}}}$ can be easily modified to accommodate a non-uniform $\Delta x$. For the non-uniform $\Delta t$, we could treat the time distance set (Fig. 1) as part of the input, to make it more flexible and specific for each data sample.

Currently, we are focusing on one-dimensional and time-independent FPE, but our method can be easily extended to higher spatial dimensions. In future work, we will try to solve the inverse problem of time-dependent FPE. Furthermore, our method can perform well when the initial noise ratio ($E_P$) is around 0.01. However, the recovered FPE terms become less accurate with data of higher noise ratio. Hence, another possible future direction is to improve the noise robustness of the proposed method.

## Supplementary Information

Supplementary Information.

## Data Availability

The data and code that support the findings of this study are available from corresponding authors upon request.
