# Supplementary material for: Solving the inverse problem of time independent Fokker–Planck equation with a self supervised neural network method
Source: Sci Rep. 2021 Jul 30;11:15540. doi: 10.1038/s41598-021-94712-5 (PMC8324819; doi:10.1038/s41598-021-94712-5)
Supplement: Supplementary file 1 — Supplementary Information. [file 41598_2021_94712_MOESM1_ESM.pdf]

Supplemental Information for
Solving the inverse problem of time independent Fokker-Planck equation with a self supervised neural network method

Wei Liu[1], Connie Khor Li Kou[1], Kun Hee Park[2], and Hwee Kuan Lee[1,3,4,5,6,7]

[1]Bioinformatics Institute, Agency for Science, Technology and Research (A*STAR), 30 Biopolis Street, #07-01 Matrix, 138671, Singapore, Singapore
[2]Centre for Quantum Technologies, National University of Singapore, 3 Science Drive 2, 117543, Singapore, Singapore
[3]School of Computing, National University of Singapore, 13 Computing Drive, 117417, Singapore, Singapore
[4]Singapore Eye Research Institute (SERI), 11 Third Hospital Ave, 168751, Singapore, Singapore
[5]Image and Pervasive Access Laboratory (IPAL), 1 Fusionopolis Way, #21-01 Connexis (South Tower), 138632, Singapore, Singapore
[6]Rehabilitation Research Institute of Singapore, 11 Mandalay Road #14-03, Clinical Sciences Building, 308232, Singapore, Singapore
[7]Singapore Institute for Clinical Sciences, A*STAR, 30 Medical Drive, 117609, Singapore, Singapore
[*]leehk@bii.a-star.edu.sg

Initializing $\hat{g}(x)$ and $\hat{h}(x)$ based on $\hat{P}^0(x,t)$ by linear least squares

In this section, $P(x,t)$, $g(x)$ and $h(x)$ are not discretized over variable x, and they are abbreviated as P, g and h for simplicity sake. We expand the right hand side of FPE:

$$\begin{aligned}\partial_t P &= \partial_x(gP) + \partial_{xx}(hP) \\ &= P\partial_x g + g\partial_x P + P\partial_{xx}h + 2\partial_x h\partial_x P + h\partial_{xx}P \\ &= (\partial_x g + \partial_{xx}h)P + (g + 2\partial_x h)\partial_x P + h\partial_{xx}P \end{aligned} \tag{1}$$

where P, $\partial_x P$, $\partial_{xx}P$ and $\partial_t P$ can be derived from P. Hence, for a fixed spatial point x and different time points $\{t_0, t_1, ..., t_n\}$, we have a matrix equation:

$$\begin{bmatrix} P_{t0} & \partial_x P_{t0} & \partial_{xx}P_{t0} \\ P_{t1} & \partial_x P_{t1} & \partial_{xx}P_{t1} \\ \vdots & \vdots & \vdots \\ P_{tn} & \partial_x P_{tn} & \partial_{xx}P_{tn} \end{bmatrix} \begin{bmatrix} \partial_x g + \partial_{xx}h \\ g + 2\partial_x h \\ h \end{bmatrix} = \begin{bmatrix} \partial_t P_{t0} \\ \partial_t P_{t1} \\ \vdots \\ \partial_t P_{tn} \end{bmatrix} \tag{2}$$

where only the three terms $\partial_x g + \partial_{xx}h$, $g + 2\partial_x h$ and h are unknown and could be easily solved by linear least squares (LLS). We then repeat the process to solve the corresponding h at all the spatial points x to derive $h(x)$ and subsequently to calculate the derivative $\partial_x h$. Finally, we can solve g from the second term $g + 2\partial_x h$. The first term is not used in this method.

As describe in the main body, we smooth P_{noisy} to derive the initialized input $\hat{P}^0$. Then we can calculate the corresponding g and h base on $\hat{P}^0$, and use them as the initialized parameters g^0 and h^0. In fact, the result of the LLS method is accurate enough only when P_{noisy} has minimal noise (Fig. S1). Otherwise, the noise will be amplified by the derivative calculations and cause significant errors in the calculated g and h. Nevertheless, as the initialized parameters, the calculated g^0 and h^0 are still more accurate than the values from the conventional initialization methods.

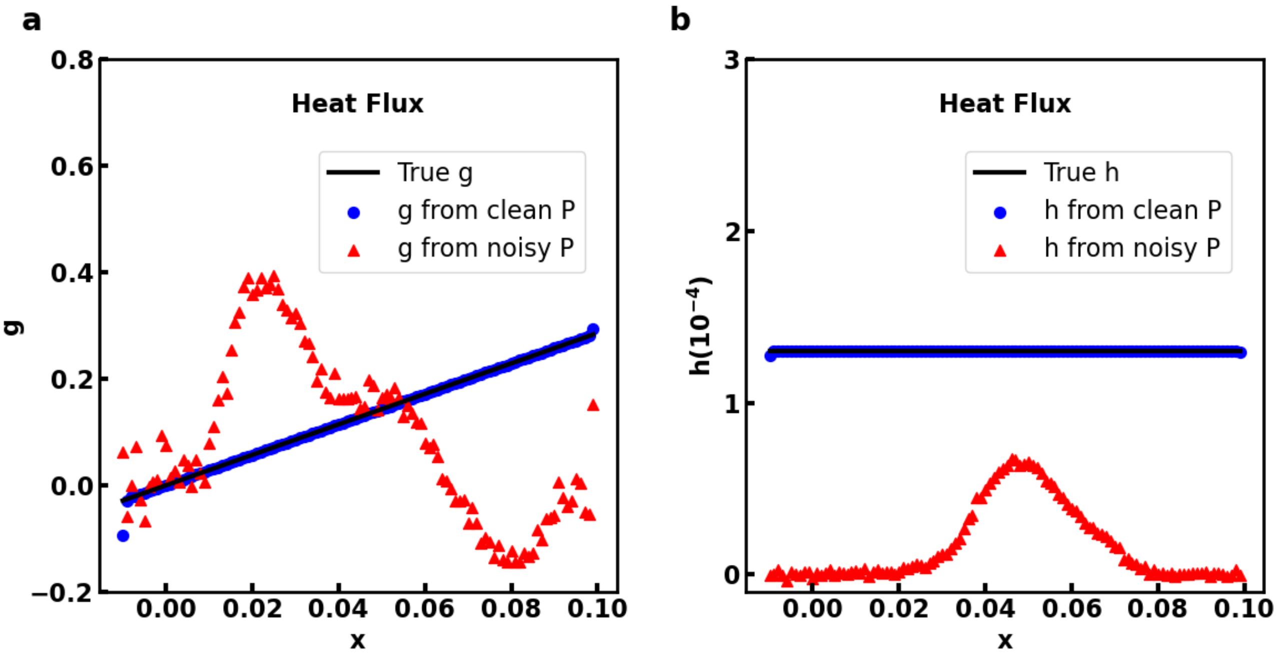

Figure S1. The calculated **g**(a) and **h**(b) using LLS with the data of the heat flux.The true FPE terms are indicated in black line —. The calculated FPE terms from the true pdf P_{clean} are indicated in ●), and the ones from the noisy pdf P_{noisy} are indicated in ▲.

Fully-connected layers to calculate derivatives with uniform grid

This method is generally a 1-dimensional version filter of the 2-dimensional filter in PDE-Net[1]. Let f be a derivable function over x, $f(x): \mathbb{R} \to \mathbb{R}$. The variable x is discretized as a vector $\boldsymbol{X} := [x_0, x_1, ..., x_m]$ with uniform gap Δx. Consequently $f(x)$ becomes a vector $\boldsymbol{F} := [f(x_0), f(x_1), ..., f(x_m)]$. To calculated the derivative of f at position x_i, we could use the Taylor series:

$$f(x_k) = f(x_i) + \frac{(k-i)\Delta x}{1!}\frac{df(x_i)}{dx} + ... + \frac{(k-i)^n \Delta x^n}{n!}\frac{d^n f(x_i)}{dx^n} + \mathcal{O}(\Delta x^n) \tag{3}$$

We substitute Eq. 3 into the dot product between $\boldsymbol{F}$ and a coefficient vector $\boldsymbol{C} := [c_0, c_1, ..., c_m]$:

$$\begin{aligned} \boldsymbol{C}\cdot\boldsymbol{F} &= \sum^{k} c_k f(x_k) \\ &= \sum^{k} c_k [f(x_i) + \frac{(k-i)\Delta x}{1!}\frac{df(x_i)}{dx} + ... + \frac{(k-i)^n \Delta x^n}{n!}\frac{d^n f(x_i)}{dx^n} + \mathcal{O}(\Delta x^n)] \\ &= A_0 f(x_i) + A_1 \Delta x \frac{df(x_i)}{dx} + ... + A_n \Delta x^n \frac{d^n f(x_n)}{dx} + \mathcal{O}(\Delta x^n) \end{aligned} \tag{4}$$

where:

$$A_n = \sum^{k} c_k \frac{(k-i)^n}{n!} \tag{5}$$

For a integer $s \leq n$, if we could find a vector $\boldsymbol{C}$ forcing A_j becomes:

$$A_j = \begin{cases} 0 & \text{if } j \neq s \quad and \quad j \leq n, \\ 1 & \text{if } j = s. \end{cases} \tag{6}$$

then Eq. 4 becomes:

$$\boldsymbol{C}\cdot\boldsymbol{F} = \Delta x^s \frac{d^s f(x_i)}{d^s x} + \mathcal{O}(\Delta x^n) \tag{7}$$

therefore:

$$\frac{d^s f(x_i)}{d^s x} = \frac{\boldsymbol{C}\cdot\boldsymbol{F}}{\Delta x^s} + \mathcal{O}(\Delta x^{n-s}) \tag{8}$$

In such a way, we could approximate the s-order derivatives of $f(x_i)$ with a high order $(n-s)$ infinitesimal error. The vector $\boldsymbol{C}$ can be solved by substituting Eq. 5 into Eq. 6. In fact, we could always find a solution of $\boldsymbol{C}$ which only has $n+1$ non-zero entries. Because the Taylor series require $(k-i)\Delta x$ to be small enough, we choose the $n+1$ entries which are closest to the i-th entry to be non-zero entries. Finally, all the coefficient vector $\boldsymbol{C}$ could be concatenated together, to generate a $m \times m$ matrix $\boldsymbol{D}$ to approximate the derivative of $\boldsymbol{F}$:

$$\frac{d^s \boldsymbol{F}}{dx^s} \approx \frac{\boldsymbol{DF}}{\Delta x^s} = \frac{1}{\Delta x^s}\begin{bmatrix} \boldsymbol{C}_{0s} \\ \boldsymbol{C}_{1s} \\ \vdots \\ \boldsymbol{C}_{ms} \end{bmatrix} \begin{bmatrix} f(x_0) \\ f(x_1) \\ \vdots \\ f(x_m) \end{bmatrix} \tag{9}$$

where $\boldsymbol{C}_{is}$ is the coefficient vector to calculate the sth-order derivative at position x_i. For example, if $m = 8$ then the matrix $\boldsymbol{D}$ to calculate the 2nd-order derivative is:

$$\begin{bmatrix} -25/12 & 4 & -3 & 4/3 & -1/4 & 0 & 0 & 0 \\ -1/4 & -5/6 & 3/2 & -1/2 & 1/12 & 0 & 0 & 0 \\ 1/12 & -2/3 & 0 & 2/3 & -1/12 & 0 & 0 & 0 \\ 0 & 1/12 & -2/3 & 0 & 2/3 & -1/12 & 0 & 0 \\ 0 & 0 & 1/12 & -2/3 & 0 & 2/3 & -1/12 & 0 \\ 0 & 0 & 0 & 1/12 & -2/3 & 0 & 2/3 & -1/12 \\ 0 & 0 & 0 & -1/12 & 1/2 & -3/2 & 5/6 & 1/4 \\ 0 & 0 & 0 & 1/4 & -4/3 & 3 & -4 & 25/12 \end{bmatrix}$$

to ensure a 3rd-order infinitesimal error for all x points. Matrix $\boldsymbol{D}$ is used as the weights of a fully-connected layer, which takes $\boldsymbol{F}$ as input and output the corresponding derivative of $\boldsymbol{F}$.

Fully-connected layers to calculate derivatives with non-uniform grid

The section shows how to generate the weights of fully-connected layers which perform derivative calculation, when there is no uniform gap Δx for discretized variable x. The whole procedure is very similar to the case with uniform grid as described previously. The major difference is that the distance between x_k and x_i cannot be represented by $(k-i)\Delta x$ and we have to use $x_k - x_i$ instead. First, we expand the function f over x by Taylor series:

$$f(x_k) = f(x_i) + \frac{x_k - x_i}{1!}\frac{df(x_i)}{dx} + ... + \frac{(x_k - x_i)^n}{n!}\frac{d^n f(x_i)}{dx^n} + \mathscr{O}((x_k - x_i)^n) \tag{10}$$

Then we multiply $\boldsymbol{F}$ which is the discretized form of $f(x)$ element-wisely to a coefficient vector $\boldsymbol{C}$:

$$\begin{aligned}
\boldsymbol{C} \cdot \boldsymbol{F} &= \sum^{k} c_k f(x_k) \\
&= \sum^{k} c_k [f(x_i) + \frac{x_k - x_i}{1!}\frac{df(x_i)}{dx} + ... + \frac{(x_k - x_i)^n}{n!}\frac{d^n f(x_i)}{dx^n} + \mathscr{O}((x_k - x_i)^n)] \\
&= A_0 i f(x_i) + A_{1i}\frac{df(x_i)}{dx} + ... + A_{ni}\frac{d^n f(x_n)}{dx} + \sum^{k} \mathscr{O}((x_k - x_i)^n)
\end{aligned} \tag{11}$$

where:

$$A_{ni} = \sum^{k} c_k \frac{(x_k - x_i)^n}{n!} \tag{12}$$

Similarly, we always can find a vector $\boldsymbol{C_i}$ forcing A_j become:

$$A_j i = \begin{cases} 0 & \text{if } j \neq s \quad \textit{and} \quad j \leq n, \\ 1 & \text{if } j = s. \end{cases} \tag{13}$$

The subscript i of $\boldsymbol{C_i}$ indicates that the vector is specific to variable point x_i. We can repeat the process to find the corresponding vector to all the other variable points, and concatenate them to form a matrix $\boldsymbol{D}$. Hence the derivative of $\boldsymbol{F}$ can be approximated by:

$$\begin{aligned}
\frac{d^s \boldsymbol{F}}{dx^s} &\approx \boldsymbol{DF} \\
&\approx \begin{bmatrix} \boldsymbol{C}_{0s} \\ \boldsymbol{C}_{1s} \\ \vdots \\ \boldsymbol{C}_{ms} \end{bmatrix} \begin{bmatrix} f(x_0) \\ f(x_1) \\ \vdots \\ f(x_m) \end{bmatrix}
\end{aligned} \tag{14}$$

Proof the integral invariance of pdfs when evolving according to FPE

Let $P(x,t)$ be a pdf which satisfies FPE. We assume $P(x,t)$ is differentiable and has a finite support $[a,b]$. Then naturally we have such boundary conditions:

$$P(a,t_0) = P(b,t_0) = 0 \qquad \left.\frac{\partial P(x,t_0)}{\partial x}\right|_{x=a} = \left.\frac{\partial P(x,t_0)}{\partial x}\right|_{x=b} = 0 \tag{15}$$

If at a time point t_0 the normalization integral is satisfied:

$$\int_a^b P(x,t_0)dx = 1 \tag{16}$$

The integral remains as 1 when the pdf evolves according to FPE:

$$\begin{aligned}
\int_a^b \frac{\partial P(x,t_0)}{\partial t} dx &= \int_a^b \left(\frac{\partial (g(x)P(x,t_0))}{\partial x} + \frac{\partial^2 (h(x)P(x,t_0))}{\partial x^2}\right) dx \\
&= \int_a^b \left(\frac{\partial (g(x)P(x,t_0))}{\partial x}\right) dx + \int_a^b \left(\frac{\partial^2 (h(x)P(x,t_0))}{\partial x^2}\right) dx \\
&= g(x)P(x,t_0)|_a^b + \left.\frac{\partial (h(x)P(x,t_0))}{\partial x}\right|_a^b \\
&= g(x)P(x,t_0)|_a^b + h(x)\left.\frac{\partial P(x,t_0)}{\partial x}\right|_a^b + P(x,t_0)\left.\frac{\partial h(x)}{\partial x}\right|_a^b \\
&= 0
\end{aligned} \tag{17}$$

Effect of the time point range in prediction when training FPE-Net with noisy target distributions

For a given x and a given time point t, Fig. S2A shows the $\boldsymbol{P}$ at current time point and nearby time points. The black triangles are $\boldsymbol{P}_{clean}$ which have a nearly linear relationship:

$$\boldsymbol{P}_{clean}(t+i\Delta t) = \boldsymbol{P}_{clean}(t) + i\Delta t \boldsymbol{\partial_t P}_{true}(t) + \mathscr{O}(i^2\Delta t^2) \tag{18}$$

The red dots are $\boldsymbol{P}_{noisy}$ which contain random noise:

$$\boldsymbol{P}_{noisy}(t+i\Delta t) = \boldsymbol{P}_{clean}(t) + i\Delta t \boldsymbol{\partial_t P}_{clean}(t) + \mathscr{O}(i^2\Delta t^2) + \boldsymbol{R}(t+i\Delta t) \tag{19}$$

where $\boldsymbol{R}(t+i\Delta t)$ is the random noise which usually is much larger than $\mathscr{O}(i^2\Delta t^2)$.

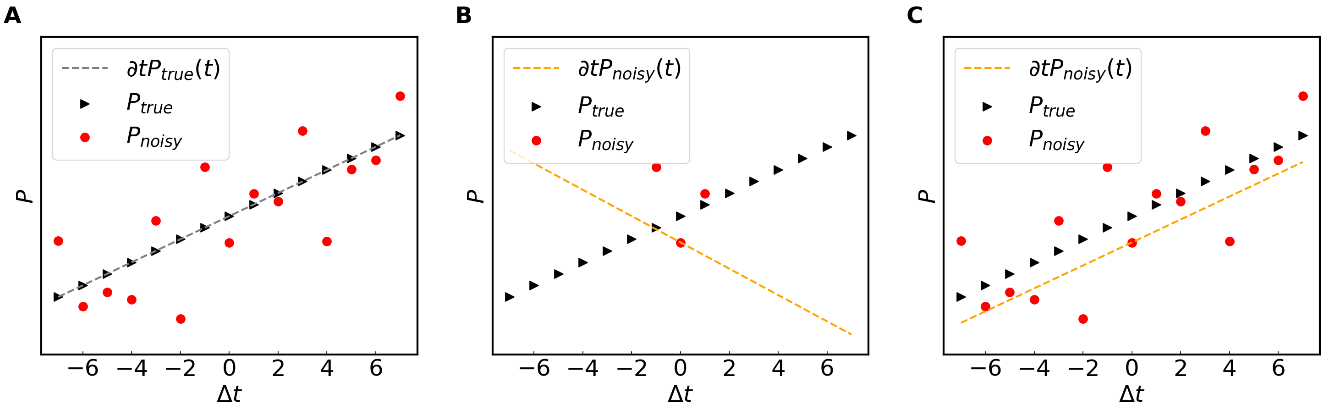

Figure S2. Plot of $\boldsymbol{P}$ and $\boldsymbol{\partial t P}$ from true or noisy data. The black triangles ▲ are $\boldsymbol{P}_{clean}$, the red circles ● are $\boldsymbol{P}_{noisy}$, the gray dash line - - is $\boldsymbol{\partial_t P}_{clean}(t)$ and the orange dash lines - - are the calculated $\boldsymbol{\partial_t P}_{noisy}(t)$.

If $\boldsymbol{P}_{clean}$ is unknown then we have to use $\boldsymbol{\partial_t P}_{noisy}(t)$ to approximate $\boldsymbol{\partial_t P}_{clean}(t)$. The $\boldsymbol{\partial_t P}_{noisy}(t)$ is derived by finding its optimal value which is used to predict:

$$\hat{\boldsymbol{P}}(t+i\Delta t) = \boldsymbol{P}_{noisy}(t) + i\Delta t \boldsymbol{\partial_t P}_{noisy}(t) \tag{20}$$

where $\hat{\boldsymbol{P}}(t+i\Delta t)$ have minimum square error with the target distribution $\boldsymbol{P}_{noisy}(t+i\Delta t)$:

$$L = \sum_{i=-n}^{n} \parallel \hat{\boldsymbol{P}}(t+i\Delta t) - \boldsymbol{P}_{noisy}(t+i\Delta t) \parallel_2 \tag{21}$$

Fig. S2B shows if we set $n=1$, the optimal $\boldsymbol{\partial_t P}_{noisy}(t)$ (orange dash line) is significantly different from $\boldsymbol{\partial_t P}_{clean}(t)$ (gray dash line) in Fig. S2A. In contrast, $\boldsymbol{\partial_t P}_{noisy}(t)$ becomes much similar to $\boldsymbol{\partial_t P}_{clean}(t)$ when we set a larger n. For example, we set $n=7$ in Fig. S2C. Hence when we train FPE NN with $\boldsymbol{P}_{noisy}$, adding more time points in prediction could help to suppress the noise effect.

A typical plot of the predicted distribution in five time gaps ahead.

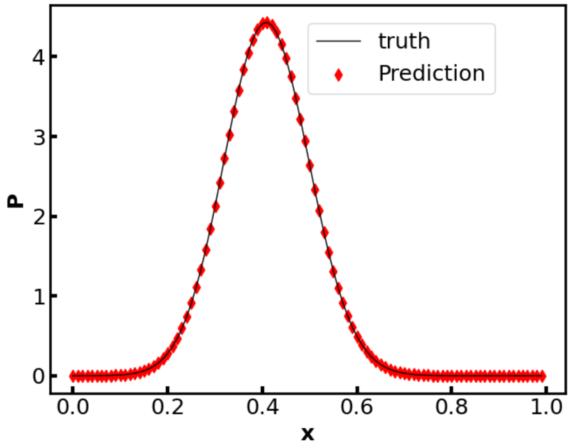

Figure S3. A typical plot of the predicted distributions in five time gaps ahead. The true distribution is shown in black line — and the predicted distribution is shown in red diamond ◆.

Summary of training results of the simulated data with different number of distribution sequences.

Example	n	distribution sequences	E_g	E_h	E_P
Flux	4	200	0.142	0.460	0.0026
Flux	4	100	0.134	0.148	0.0028
Flux	4	75	0.395	0.310	0.0028
Flux	4	50	0.787	0.676	0.0030
Bubble	8	200	0.265	0.063	0.0016
Bubble	8	100	0.390	0.043	0.0014
Bubble	8	75	0.455	0.091	0.0011
Bubble	8	50	0.638	0.330	0.0012
Wealth	4	200	0.124	0.136	0.0018
Wealth	4	100	0.110	0.061	0.0018
Wealth	4	75	0.626	0.457	0.0018
Wealth	4	50	0.836	0.459	0.0018

Table S1. Summary of the training results of the simulated data from three FPE examples, with different number of distribution sequences. n is the output time point range and we chose the one with the best result when training with 100 distribution sequences.

The training results of the simulated data with higher initial noisy ratio (E_P)

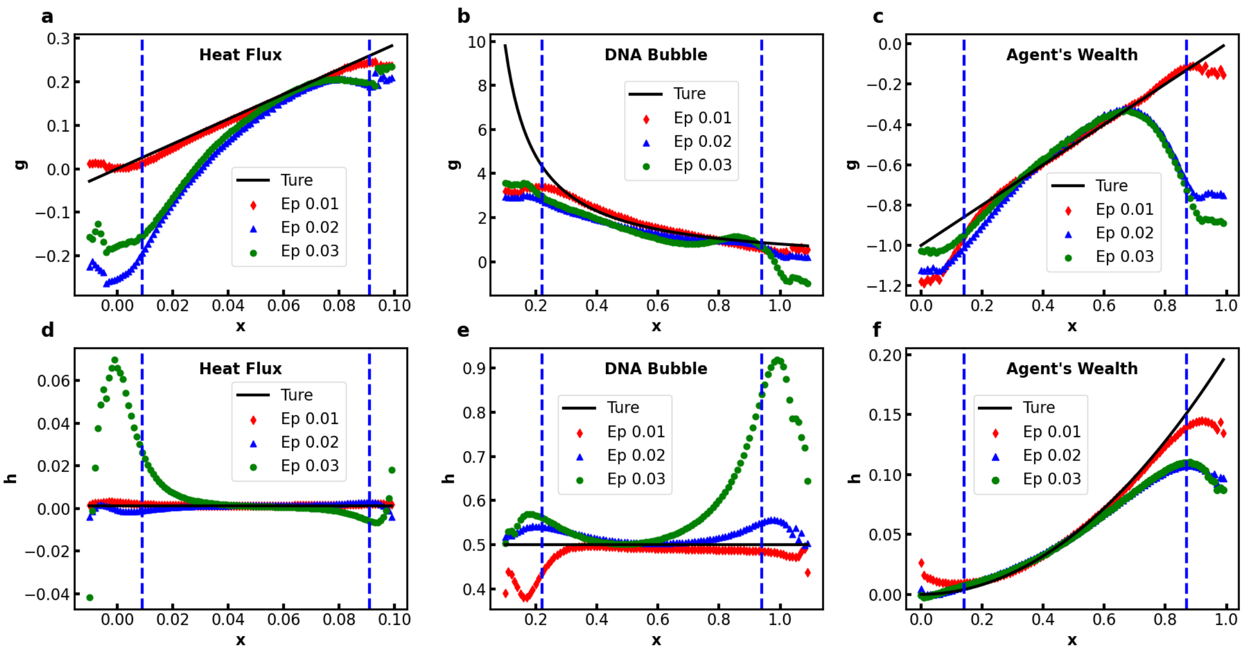

Figure S4. The final training result of the simulated data with initial noise ratio (E_P) 0.01 (red diamond ◆), 0.02 (blue triangle ▲), and 0.03 (green circle ●). The boundary area is indicated by the vertical blue dash line. All the training processes use the same hyperparameter $n = 8$. The true $\boldsymbol{g}$ and $\boldsymbol{h}$ are indicated in black line —. The result that the calculated $\hat{\boldsymbol{g}}$ and $\hat{\boldsymbol{h}}$ fit their true values very well, but a higher noise ratio could cause the training result less accurate.

Validation of FPE-NN on FPE for Brownian motion in a periodical potential

We validate our method on another FPE example, the Brownian motion in a periodic potential which is applicable in many field[2–5]. In our experiment, the system consists of a sinusoidal potential with a constant external force. The total potential is $V(x) = -0.4cos(0.2x) - 0.002x$ (Fig. S5a), where x is the random variable with support of $[-11\pi, 11\pi]$. The corresponding FPE for the Brownian motion on the potential is:

$$\frac{\partial P(x,t)}{\partial t} = \frac{\partial}{\partial x}(P(x,t)\frac{\partial V(x)}{\partial x}) + \frac{\sigma^2}{2}\frac{\partial^2 P(x,t)}{\partial x^2} \tag{22}$$

where we set the diffusion coefficient as $\sigma = 0.3$. The simulated data is generated following the same way as the three FPE examples in the main text. The time gap Δt is adjusted to be 1. The train result table and the plot of trained FPE terms are shown below, suggesting our model can perform well when the FPE term has a complex non-polynomial form.

n	E_g	E_h	$\tilde{E}_g$	$\tilde{E}_h$	E_P
2	1.349	0.007	0.069	0.018	0.0023
4	1.363	0.009	0.112	0.047	0.0025
6	1.385	0.011	0.137	0.058	0.0038
8	1.640	0.011	0.123	0.028	0.0064

Table S2. Summary of the training results of the simulated data from the Brownian motion in a periodic potential, with 100 distribution sequences. n is the output time point range used in Eq. 4 and Eq. 6 on page 5. E_g, E_h and E_P are the error metrics as defined in Eq. 11 on page 6. $\tilde{E}_g$ and $\tilde{E}_h$ are the corresponding metrics when the boundary area is ignored.

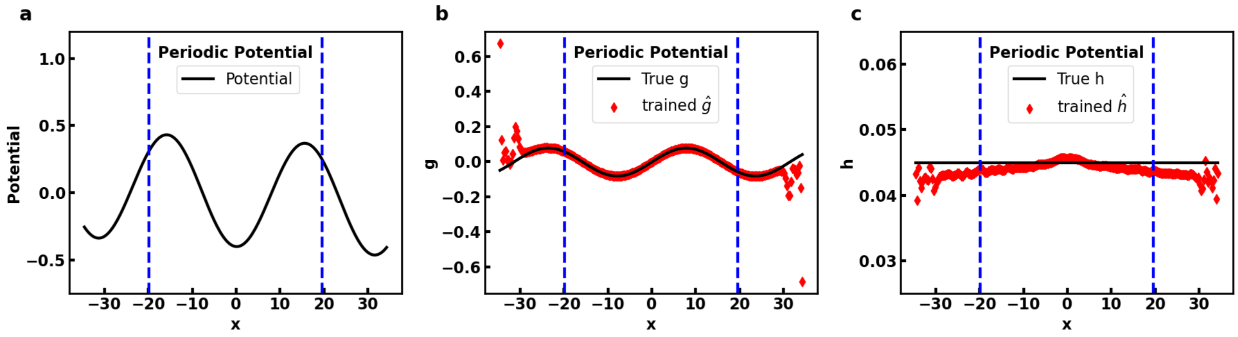

Figure S5. Final training result of the simulated data from the Brownian motion in a periodic potential, with $n = 2$. The boundary area is indicated by the vertical blue dash line. (a) The periodic potential. (b) The true $\mathbf{g}$ (black line —) and the calculated $\hat{\mathbf{g}}$ (red diamond ◆). (c) the true $\mathbf{h}$ (black line —) and the calculated $\hat{\mathbf{h}}$ (red diamond ◆).

References

1. Long, Z., Lu, Y., Ma, X. & Dong, B. Pde-net: Learning pdes from data (2018). 1710.09668.
2. Fulde, P., Pietronero, L., Schneider, W. R. & Strässler, S. Problem of brownian motion in a periodic potential. *Phys. Rev. Lett.* **35**, 1776–1779, DOI: 10.1103/PhysRevLett.35.1776 (1975).
3. Josephson, B. Possible new effects in superconductive tunnelling. *Phys. Lett.* **1**, 251–253, DOI: https://doi.org/10.1016/0031-9163(62)91369-0 (1962).
4. Ambegaokar, V. & Halperin, B. I. Voltage due to thermal noise in the dc josephson effect. *Phys. Rev. Lett.* **22**, 1364–1366, DOI: 10.1103/PhysRevLett.22.1364 (1969).
5. Dieterich, W., Peschel, I. & Schneider, W. R. Diffusion in periodic potentials. *Zeitschrift für Physik B Condens. Matter* **27**, 177–187, DOI: 10.1007/BF01313607 (1977).
